# Smartwatch assessment of CPR performance administered to an instrumented mannequin

**DOI:** 10.1007/s11517-026-03533-z

**Published:** 2026-03-03

**Authors:** Edilson Fernando de Borba, André Luiz Felix Rodacki, Gustavo Hoffmann, Sara Batista Honorato, Luis Henrique Gabira Perez, John Gerard Buckley, Anderson Zampier Ulbrich

**Affiliations:** 1 Departamento de Educação Física, Setor de Ciências Biológicas, Centro de Estudos do Comportamento Motor (CECOM/UFPR), Universidade Federal do Paraná (UFPR), Curitiba, PR Brasil; 2 Departamento de Medicina Integrada, Centro de Ciências da Saúde, Grupo de Pesquisa em Medicina do Exercício (MedEx/UFPR), Universidade Federal do Paraná (UFPR), Curitiba, PR Brasil; 3 Faculty of Engineering & Digital Technologies, University of Bradford, Bradford, BD7 1DP West Yorkshire England, UK

**Keywords:** Cardiopulmonary resuscitation (CPR), Smartwatch sensors, Real-time feedback, CPR training

## Abstract

Cardiac arrest is a life-threatening emergency that requires effective cardiopulmonary resuscitation (CPR) to preserve life. Smartwatches equipped with accelerometer sensors and worn close to the wrist joint may offer an accessible way to provide CPR feedback. This study determined whether accelerometer data from a smartwatch could provide valid estimates of CPR parameters, including compression, decompression, frequency, and cycle time. Nineteen medical school students performed CPR on an instrumented mannequin while wearing a smartwatch. CPR parameters were derived from the smartwatch’s accelerometer signals using a custom algorithm after first resampling the data to 60 Hz to circumvent the issue of small fluctuations in the sampling frequency inherent in these devices. Results were compared with kinematic data obtained from video analysis undertaken simultaneously. Across 3,005 CPR cycles, smartwatch analysis indicated that 76.7% of compressions were within the recommended depth range (5–6 ± 0.5 cm), while the mannequin software reported a mean performance score of 98.7 ± 1.35%. There was good agreement between smartwatch and video analysis for cycle duration (bias = 0.00 s; 95% CI: −0.05 to 0.05 s) and compression depth (bias = − 0.31 cm; 95% CI: −1.61 to 0.98 cm). These findings demonstrate that smartwatch accelerometer data analyzed with the proposed algorithm can accurately assess CPR performance administered to a mannequin, supporting its potential use as a practical assessment tool for CPR. Further work is needed to develop a smartwatch App that provides real-time feedback to improve CPR performance during training and real-world resuscitation scenarios.

**Graphical Abstract:**

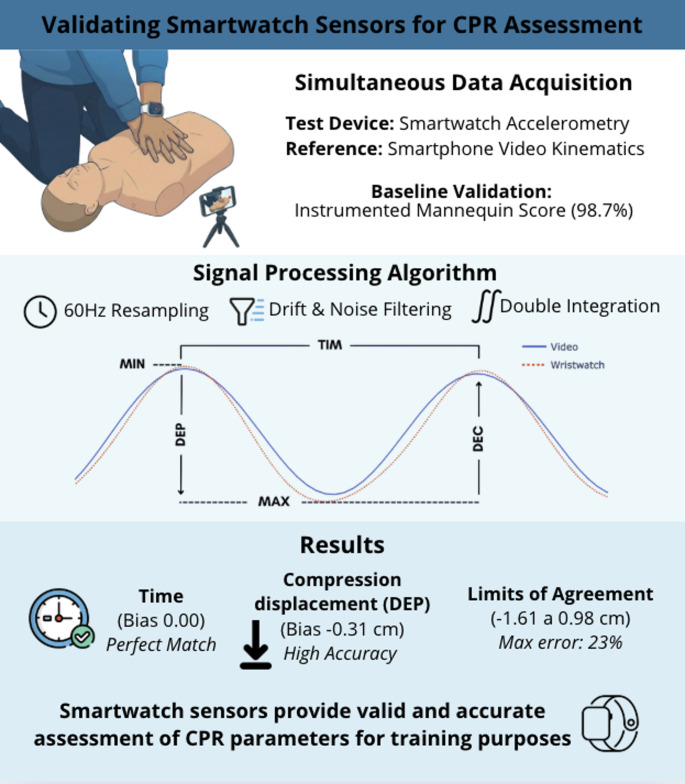

**Supplementary Information:**

The online version contains supplementary material available at 10.1007/s11517-026-03533-z.

## Introduction

 A cardiac arrest is a critical cardiovascular event predominantly related to the loss of heart function leading to cessation of blood circulation [1]. In the United States, the incidence of cardiac arrest is estimated to be one event every 90 s, totaling approximately 356,000 adults with a cardiac arrest event per year [2]. In Brazil, approximately 400,000 people died in 2022 from a cardiovascular episode [3]. Interestingly, most events occurred outside a hospital, of which 71.3% were at home, 18% in the public setting, and 10.7% in a nursing home facility [4]. Survival chances from a cardiac arrest are two to three times greater when a rapid response that includes cardiopulmonary resuscitation (CPR) is administered whilst waiting for medical assistance [5]. Those who receive CPR have greater survival rates than those who did not receive CPR (18.3 vs. 7.1%, respectively) [6].

The CPR recommendations from the American Heart Association [1] and the European Resuscitation Council [7] include using auxiliary feedback from audiovisual devices to optimize CPR performance. It has been demonstrated that CPR efficiency depends on devices with instructional resources involving feedback and debriefing, contextualized learning, practice stimuli, and innovative strategies [8]. The ubiquity of smartphones has favored the use of applications to provide either visual (e.g., movement depth information), audio (e.g., frequency control information), or tactile (e.g., vibration information to control the CPR pace) feedback. Despite a large number of applications developed for smartphones [9], their widespread use is inhibited because rescuers/first aiders cannot hold them during CPR [10]. The use of wearable sensors embedded in smartwatches may offer an alternative means to monitor CPR performance. As a smartwatch’s sensors will be close to the wrist joint, their use offers the potential to provide real-time feedback on how well the CPR is being administered.

Current CPR feedback systems are typically expensive, require specialized equipment, or are not readily available for real-world applications, limiting their widespread use in both training and emergency care. Smartwatches, on the other hand, are affordable, widely adopted, and equipped with built-in motion sensors that could enable accessible monitoring of CPR quality.

Therefore, this study aimed to determine whether data from the accelerometer sensors embedded in a smartwatch can be used to monitor the number of cycles, compression depth, and pacing of CPR performed on a mannequin. If the algorithm accurately measures these parameters, it may provide real-time feedback on cycle frequency and compression depth through the manufacturer’s QCPR App.

A novel method to estimate displacement from acceleration signals from a smartwatch, is to resample the acceleration signals to eliminate the small fluctuations in the sampling frequency that are inherent in such devices. This resampling is done on a cycle-by-cycle basis to avoid the potential issue of ‘drift’, before displacement is then calculated using a double integral approach. If this innovative approach can assess CPR performance accurately, it may enhance measurement precision and demonstrate the potential of smartwatches in supporting emergency care as a possible feedback tool. The proposed algorithm integrates signal-processing refinements seldom considered in previous CPR feedback studies, including correction of timestamp irregularities, interpolation to stabilize sampling frequency, and cycle-by-cycle recalibration to minimize drift accumulation. These procedures are expected to improve the reliability of displacement estimation and provide a more stable reconstruction of CPR compression and decompression patterns over time.

## Methods

Nineteen medical school students (24.3 ± 5.4 years; 67.4 ± 9.3 kg; 1.7 ± 0.1 m) were invited and agreed to participate. All participants had completed a first aid (training and simulation) course, which included being taught CPR procedures, within the 3 months preceding their participation in the study. Participants were physically able and had no musculoskeletal limitations that could impede or restrict CPR application. The study was conducted in accordance with the Declaration of Helsinki, and the data was collected between May and July 2024. All participants signed a participation consent form approved by the Ethics Committee from the Clinics Hospital of the Paraná Federal University (CAAE 30108420.30000.5225).

Participants received verbal instructions of the protocol along with a brief demonstration of CPR applied to the mannequin and were given two CPR familiarization trials before their CPR performance was analyzed. They were instructed to perform CPR according to the American Heart Association guidelines [1]. They assumed a comfortable kneeling position perpendicular to the instrumented mannequin (*Little Anne QCPR*,* Laerdal Medical*,* Model 120-60750*) sagittal plane, with their hands positioned at the mammary line. The mannequin’s sensors were paired to a smartphone (iPhone 11, iOS 17), and the performance was monitored on a cycle-by-cycle basis (real-time) using the manufacturer’s QCPR App/software. Through this App, they received real-time audiovisual feedback regarding compression depth and CPR rate. They applied CPR for 2 min in two bouts (T1 and T2), with a rest interval of 2 min between bouts. The 2-minute rest simulated what would occur when swapping between first aiders (which is recommended in CPR guidelines).

**Fig. 1:**
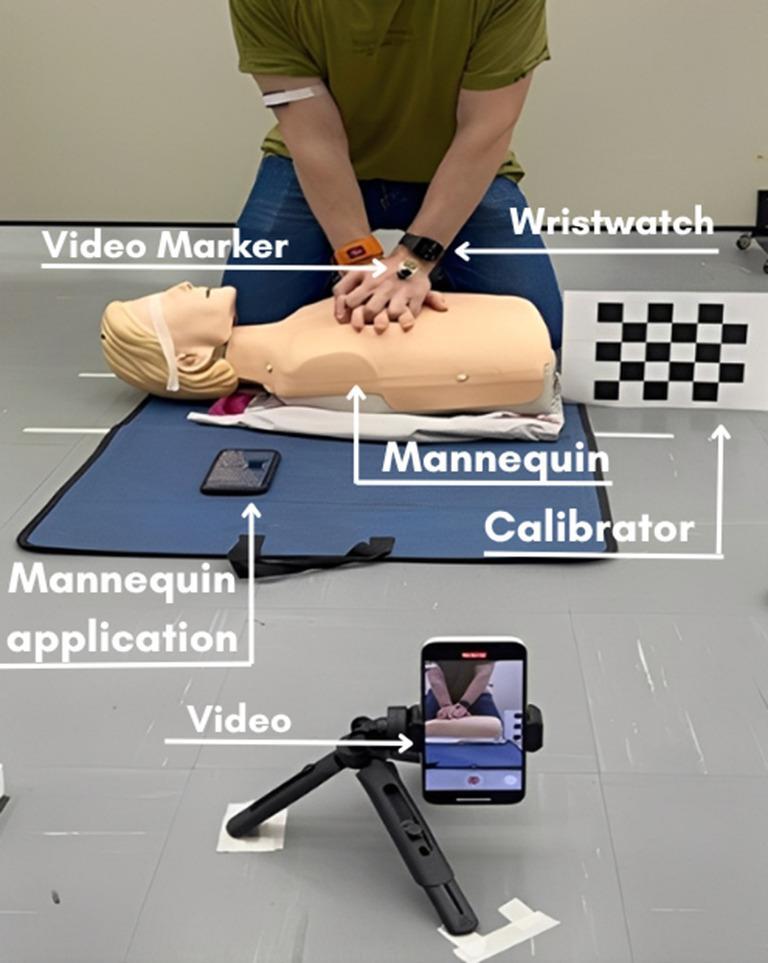
The data collection setup with the participant kneeling at the side of the instrumented mannequin while applying RCP. The smartphone used for capturing the video, the smartwatch, and the marker placed on the hand are indicated

Participants wore a smartwatch (*Apple Watch Series 5 44 mm*, *WatchOS 10.5*) attached (snugly) to their left wrist, paired with a second smartphone (*Apple iPhone 13*, *iOS 17*). The watch’s resultant acceleration data series were recorded using the ForceData application (*ForceData*,* USA)*, sampling at 200 Hz. The data were transferred to a PC for further processing. Figure 1 illustrates the experimental setup.

Although the mannequin provides real-time audiovisual feedback, it does not generate, or store detailed (cycle-by-cycle) compression data for subsequent analysis. Consequently, no compression depth excursion or other metrics were available when the CPR sessions ended. The only recorded output provided by the mannequin’s App/software was a CPR ‘performance’ score. Therefore, to assess the accuracy of the CPR compression depth and frequency measures determined from the analysis of the smartwatch’s acceleration data, the measures were compared to compression depth and frequency measures determined by video analysis. The video analysis was undertaken simultaneously using the following procedures. Each 2-minute CPR bout was recorded using a smartphone’s video camera (*iPhone 12*, *iOS 17*), sampling at 60 Hz. The smartphone was fixed on a tripod positioned, approximately 1.5 m away, perpendicularly to each participant’s frontal plane (Fig. 1). The camera was centered on the wrist joint with a field of view of approximately 4–5 cm. A calibration object (a grid of 4 × 5 squares of 3.5 × 3.5 cm each) was placed in the plane of motion. The videos were analyzed using the software Kinovea (version 2023.1.1), and subsequently, the vertical position of a marker (0.25 cm in diameter) positioned on the dorsum of the hand (over the middle of the capitate, in the line of the third finger) was tracked using the software’s automatic recognition tool. The time-series marker displacement data were analyzed to identify the compression depth (i.e., the downward vertical linear displacement of the marker) and decompression displacement (i.e., the upward vertical linear displacement of the marker) of each cycle, the cycle frequency, and the number of cycles completed.

The kinematic analysis procedures used in the present study have been shown to have high intra- and inter-rater reliability (ICC > 0.98; [11]). All trial bouts performed by the participants achieved a performance score (highlighted by the QCPR App) above 93.0%, and hence, all bouts were selected for subsequent analysis.

## Data treatment

The time-series resultant acceleration data from the smartwatch were filtered using a 2nd order low-pass Butterworth Filter with a cutoff frequency of 3 Hz. Then, the time vector of the accelerometer data series was resampled to 60 Hz, with data being interpolated using a cubic spline function to minimize the effects of any small oscillations in time [12]. This procedure was undertaken because we had noticed the smartwatch’s sampling frequency was slightly inconsistent (i.e., it ranged from 58 to 62 Hz). The acceleration signal was then integrated twice to obtain displacement. The integration procedure used the median acceleration (calculated from the entire signal) to centralize the signal baseline to avoid ‘drifting’. The integration procedure included a high-pass Butterworth filter with a cutoff frequency of 0.01 Hz and a Tukey window of 0.1% of the signal length.

**Fig. 2:**
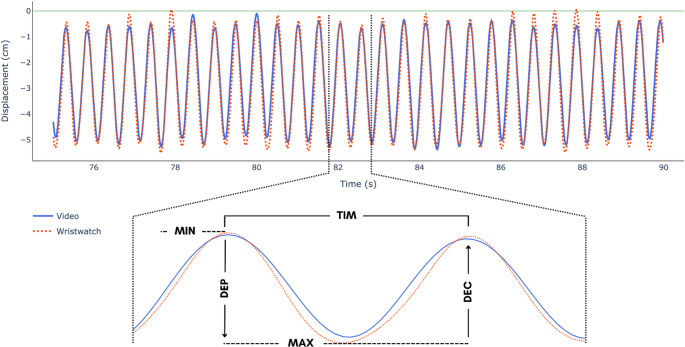
Compression depth estimated by the smartwatch and video analysis (upper panel) and a schematic representation of the key instants of a typical cycle. DEP = compression displacement; DEC = decompression displacement; TIM = cycle duration; MIN = no compression; MAX = maximum compression excursion

By applying a gentle tap on the smartwatch’s screen at the beginning of the recording of the CPR assessment, which was also recorded by the smartphone’s camera, the smartwatch and the video data series were retrospectively time-synchronized by making the recorded ‘tap’ in each recording modality ‘time zero’. However, due to the resampling of the smartwatch’s accelerometer data (described above) potentially introducing a small time offset in comparison to the recorded video data, a cross-correlation function with a 15-point window (250ms) was subsequently applied to ensure there was zero lag between the data series from each measurement modality (smartwatch, video). The time-series vertical displacement signals from each recording modality were then analyzed in the following way: a “find peaks” function identified each CPR cycle’s minimum and maximum vertical displacement; the distance between the minimum and maximum displacement defined each CPR cycle’s compression depth (DEP), while the distance between the maximum and minimum displacement defined the cycle’s decompression (DEC). The time between the instant of each successive positive (minimum compression) peak relative to the instant of the preceding positive peak determined the cycle duration (TIM). The number of positive peaks identified defined the total number of cycles (TOT). The pacing of the CPR cycles was recorded as the number of cycles per second (FRQ). Figure 2 presents a schematic representation of the time-series displacement signals determined from the smartwatch and video assessment, along with the variables analyzed. All data processing procedures were performed using customized scripts in *Python (version 3.12)*.

## Statistics

To evaluate whether analysis of the smartwatch’s accelerometer signal can provide a valid assessment of CPR parameters, the agreement between the output parameters (i.e., DEP, DEC, and TIM) determined from analysis of the smartwatch’s time series acceleration signal and those determined from analysis of the video was assessed using Bland-Altman analysis [13]. This analysis was performed using data from all CPR cycles (i.e., bouts T1 and T2; *n* = 3005 cycles). For the present analysis, the data from 19 participants were deemed sufficient to produce sufficient data variability to ensure that readings were made over a relatively extensive range of depth and frequencies across 3005 CPR cycles (e.g., shallow, deep, fast, and slow). All cycles were considered, and there was no missing (video or smartwatch) data. The statistical analyses were performed using the JASP software (version 0.18.3).

## Results

As indicated by the mannequin manufacturer’s App/software, the group-mean CPR ‘performance’ score achieved during the CPR procedures was 98.7 ± 1.35%. The smartwatch assessment indicated that 76.7% of 3005 CPR cycles performed by participants (there were no missing data) were performed within the recommended depth range (i.e., 5–6 ± 0.5 cm). We included the ± 0.5 cm to take into account movement artifact, e.g., of the watch on the wrist and/or due to hand deformation during the compression movement. Table 1 presents the group average and coefficients of variation (CV) for the various CPR parameters determined via smartwatch analysis and video analysis. It is apparent that when administering CPR, participants were more consistent in controlling the temporal aspects (cycle time and frequency; CV ≤ 5.6%) in comparison to the spatial aspects (compression and decompression; CV ≤ 15.6%).

**Table 1:** The average (± standard deviation) of the parameters of the CPR obtained from the smartwatch sensors and video kinematics (video)

TOT (*n*)	Smartwatch	CV%	Video	CV%	Difference (%)
	3005	-	3005	-	0 (0)
DEP (cm)	5.56 ± 0.98	17.6	5.25 ± 0.82	15.6	0.31 (5.9%)
DEC (cm)	5.56 ± 0.97	17.4	5.25 ± 0.82	15.6	0.31 (5.9%)
TIM (s)	0.53 ± 0.02	3.8	0.53 ± 0.03	5.6	0 (0)
FRQ (Hz)	1.88 ± 0.09	4.8	1.88 ± 0.09	4.8	0 (0)

Difference (%) = Difference between smartwatch and video and relative difference; CV% = coefficient of variation; TOT= number of cycles, DEP= compression displacement, DEC = decompression displacement, TIM = cycle duration, and FRQ= cycle pacing

When the parameters determined from the assessment of the smartwatch’s sensors were compared to those determined from video analysis, the number of cycles was identical, and the average duration of the cycles was also coincident (see Supplementary Material, S1 Table). The mean values of each of the parameters determined by both measurement approaches are presented in Table 1.

**Fig. 3:**
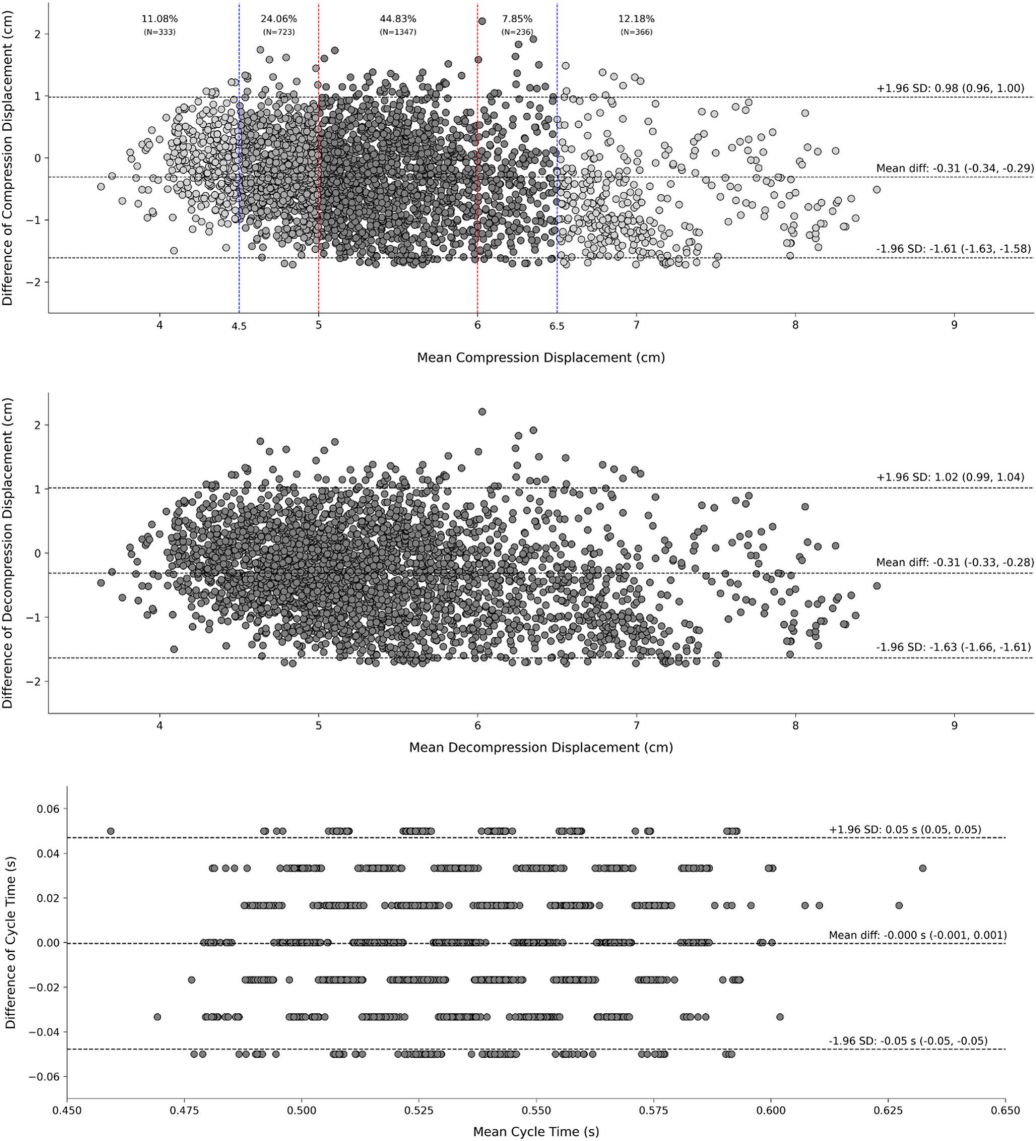
Bland-Altman agreement analysis for the compression depth (DEP; upper panel), decompression (DEC; middle panel), and cycle time (TIM; lower panel) for all participants (*n* = 3005 cycles). The vertical red dashed lines represent the compression range recommended by the American Heart Association, and the vertical blue dashed lines indicate the recommended range but with an additional ± 5 mm to account for measurement errors. The percentage of the cycles within the different ranges are indicated. The upper and lower horizontal lines represent the 95% limits of agreement, while the central horizontal line represents the mean difference between the two measurements

There was good agreement between the cycle duration determined from the smartwatch analysis and that from the video analysis (TIM: bias < 0.001s; Limits of Agreement 95%: -0.05s, 0.05s). Consequently, the determined frequency at which the cycles were performed (1.88–1.88 Hz) was coincident across measurement modalities. There was also good agreement between the compression depth and decompression determined from the smartwatch analysis and that from the video analysis (DEP: bias = − 0.31 cm; Limits of Agreement: 95% − 1.61, 0.98 cm; DEC: bias = − 0.31 cm; Limits of Agreement 95%: − 1.63, 0.102 cm). Figure 3 shows the Bland-Altman agreement for the DEP, DEC, and TIM.

## Discussion

The higher agreement observed between smartwatch-derived CPR parameters and those determined by video analysis, compared to previous reports, may be due to algorithmic refinements implemented prior to integrating the smartwatch’s acceleration signal. The key refinements were the correction of timestamp irregularities and the cycle-by-cycle recalibration procedure, which have not been systematically applied in previous work using inertial sensors to assess CPR parameters. Although the idea of using smartwatches is not novel, there is still a need to develop data-processing approaches to achieve more accurate identification of CPR parameters from the smartwatch’s acceleration signal. The current study indicated that analysis of a smartwatch’s accelerometer data can attain estimates of the number of cycles, compression depth and decompression, frequency, and cycle duration of CPR, and can do this with comparable precision to video analysis. In addition, it shows that the proposed algorithm provides a mean compression depth error (e.g., 0.31 cm; 5.9%) that is smaller (~ 10% [14]) or comparable (~ 6% [15]) to previous reports.

As well as the mean compression depth error/bias being smaller (i.e., it is more accurate) than reported in previous studies, the present analysis also extends beyond simply providing an estimation of compression depth bias by providing additional parameters: compression displacement (DEP), decompression displacement (DEC), cycle duration (TIM), and pacing (FRQ).

Methodologically, inertial-sensor approaches for assessing CPR performance may be classified onto two families: (i) Integration-based algorithms that derive CPR compression displacement (DEP) by double integration of the gravity-compensated tri-axial acceleration signal (typically the vector magnitude) and then extracting the peak-to-peak depth per cycle. The usual steps in this approach include resetting constants on a per-cycle basis to control for drifting, paired low-high-pass smoothing [9, 14, 15], and refinements such as explicit orientation correction and band-pass filtering [16]. Sampling rate is not constant, and most works do not perform timestamp resampling, which may contribute to lower accuracy; (ii) Model-based algorithms predict DEP from acceleration energy (e.g., mean squared acceleration over ~ 3 s) and sampling rate via polynomial fitting, thereby avoiding the need for signal integration but does require device-specific calibration and using sampling rate as a covariate [10]. Table 2 summarizes the main computational pipelines reported for inertial-sensor–based CPR assessment, highlighting substantial heterogeneity in both signal processing choices (e.g., integration-based versus model-based estimation, drift/noise handling, and cycle segmentation) and validation procedures across the literature. Most studies have been validated on instrumented mannequin using displacement ground truth (typically LVDT or dedicated displacement sensors) and frequently report performance using absolute error or range-based summaries, whereas fewer works provide agreement statistics such as Bland–Altman bias and limits of agreement. In addition, published methods often prioritize compression depth (DEP) and/or rate, with limited reporting of decompression displacement (DEC), cycle duration (TIM), or complete cycle-by-cycle extraction details. Consequently, any state-of-the-art comparison must explicitly state (i) the reference system, (ii) the definition of error (absolute error versus signed bias/limit of agreement), and (iii) which CPR metrics are produced by each method to ensure that observed differences reflect method performance rather than differences in experimental set-up or reporting conventions.

**Table 2:** Computational pipelines used for inertial sensor–based CPR assessment

Study (year)	Device	Reference System	Core Method	Noise and Drift Handling	Cycle Detection
Song et al., 2015 [9]	Smartphone (U-CPR; 3-axis accel.)	LVDT on Manequim (1 kHz)	Double integration per-axis; CCD via vector sum per cycle; reset constants	Weighted smoothing (LP-like) + transient emphasizing (HP-like)	Peak-to-peak on displacement for DEP
Jeong et al., 2015 [12]	Smartwatch (Galaxy Gear Live; 3-axis accel.)	LVDT on Mannequin (Little Anne)	Double integration per-axis; CCD via vector sum	Low-pass for noise + high-pass for gravity/drift (recursive smoothing)	Peak-to-peak displacement for DEP
Song et al., 2016 [11]	Smartphone (Galaxy S3) & Smartwatch (Gear Live)	RDP-100 S displacement sensor inside a Little Anne manikin	Double integration of 3-axis acceleration (algorithm from prior smartphone work)	Weighted smoothing (LP-like) + transient emphasizing (HP-like)	Per-cycle depth vs. reference; DEP error
Lu et al., 2018 [10]	Smartwatch (ASUS ZenWatch 2)	Resusci Anne QCPR + PC Skill Reporting	Polynomial model: CCD_pred = f(M, rate), M = mean of squared accel over T=3s	Gravity removal by projecting a on g; model-based (no explicit timestamp correction)	Per-sample prediction via f(M, rate); DEP bias via BA
Lee et al., 2021 [16]	Smart-Ring (IMU 9-axis, MPU-9250)	LVDT (100 Hz), synchronized with ring	Double integration per axis after gravity/orientation removal; vector magnitude	Butterworth HPF ~ 0.5 Hz (remove DC) + LPF 3rd order (denoise); Orientation (Euler angles) + rotation matrix	Min–max–2nd min per cycle; DEP as mean peak-to-peak; DEC NR; moving average of 5 cycles
Present study	Apple Watch S5 (accelerometer)	Video kinematics 60 Hz	Double integration of corrected acceleration; cycle-by-cycle recalibration	Resampling 58–62→60 Hz (cubic spline), median centering, Tukey window, HPF 0.01 Hz, LPF 3 Hz	Find peaks on displacement: DEP = max–min; DEC = min–max; TIM from minima; FRQ from cycles/s

Summary of computational approaches reported in the included inertial-sensor CPR studies. Columns describe the device/sensor setup, reference system, core estimation strategy (e.g., double integration vs. model-based prediction), noise/drift mitigation (filtering and gravity/orientation handling), and the procedure used to segment cycles and extract CPR metrics. A complementary table provides the state-of-the-art performance comparison (errors/limits of agreement) for DEP, DEC, TIM, and FRQ when available

The signal processing pipeline in the present study used double integration, timestamp resampling (58–62 → 60 Hz via cubic spline), median centering, Tukey windowing, a high-pass filter at 0.01 Hz, a low-pass filter at 3 Hz, and cycle-by-cycle recalibration. This approach enabled robust reconstruction of both CPR compression and decompression phases and, hence, provided assessment of DEP, DEC, TIM, and FRQ, CPR elements that are absent or partially addressed in previous studies.

Table 3 summarizes the CPR performance metrics (DEP, DEC, TIM, FRQ, and LoA) across previous studies, with metrics from the present one included for comparison. It shows that previous studies have used 1 or 2 parameters, whereas the present study quantified all 4. Reporting multiple metrics may help clarify methodological trade-offs and is more informative for algorithm development and eventual translation to training or real-world use. In contrast, using only a single parameter (e.g., mean depth error) may mask temporal inaccuracies or asymmetrical cycles.

**Table 3:** Comparison of inertial-sensor–based methods for CPR assessment

Study	Mean Error Dep (cm)	Dep LoA (cm)	Mean Error Dec (cm)	Dec LoA (cm)	Mean Error Tim (s)	Mean Error Frq (CPM)
Song et al., 2015 [9]	0.14 to 0.31	NR	NR	NR	NR	NR
Jeong et al., 2015 [12]	0.25 to 0.38	NR	NR	NR	NR	NR
Song et al., 2016 [11]	0.23 to 0.53	NR	NR	NR	0.02	NR
Lu et al., 2018 [10]	0.00	−0.08 to 0.09	NR	NR	NR	NR
Lee et al., 2021 [16]	0.14 to 0.23	−0.46 to 0.05	NR	NR	NR	±1
Present study	0.31	−1.61 to 0.98	0.31	−1.63 to 0.102	0.00	0.00

Assessment metrics from the present study in comparison to published inertial-sensor–based methods: compression displacement (DEP), decompression displacement (DEC), cycle duration (TIM), and cycle pacing (FRQ). Mean errors and limits of agreement (LoA) are presented when available. NR = not reported

The limits of agreement for compression depth indicate that the two measurement methods (smartwatch and video analysis) can be expected to disagree by up to 1.3 cm in either direction, which represents approximately 23% of the mean depth (i.e., 5.56 cm). Consequently, 95% of the measurements obtained from the smartwatch may range between 4.2 and 6.8 cm when compared with video analysis. Although this apparent inaccuracy exceeds the target compression range (5.0–6.0 cm), it should be emphasized that video analysis may not represent the absolute “ground truth” of chest displacement. Future studies should directly measure the vertical deformation of the mannequin’s chest using a Linear Variable Differential Transducer (LVDT) as the reference method. Such work is needed to establish whether smartwatches can provide sufficiently reliable feedback for both training and real-world resuscitation settings.

From a practical perspective, the observed variability in compression depth (± 1.3 cm) warrants caution. Although this accuracy level is comparable to other wrist-worn or smartphone-based systems [14, 17], it remains insufficient for clinical-grade feedback, where the acceptable range is narrow ± 0.5 cm (i.e., compression depth should be 5.5 ± 0.5 cm). Nevertheless, this precision may still be adequate for training or educational contexts, where the goal is to guide general technique and rhythm rather than provide exact numerical feedback.

It is unclear why the cpr scores provided by the mannequin software were given a ‘performance’ score of, on average, 98.7%, whilst the smartwatch analysis indicated that only ~ 77% of the CPR cycles were performed to within the recommended compression depth. We were unable to gain any information from the mannequin manufacturer about the algorithm used to determine CPR ‘performance’ scores. Therefore, we are unable to provide any clarity regarding what a cpr score of 98.7% indicates, i.e., if and how aspects of consistency in compression depth and cycle frequency are included in the CPR ‘performance’ score. In obtaining such a ‘high’ cpr score, it would seem that other parameters, such as ventilation scores (i.e., ventilation volume, ventilation rate, number of pre-ventilations, inspiration time in pre-ventilations) and flow fraction (i.e., percentage of the time where compressions were given), are computed in addition to the compression score to determine the overall score [18]. Thus, obtaining a clear measure of the compression scores from the output of the mannequin’s software would seem problematic. However, the proportion of cycles within the adequate range of the present study (44.8%) is similar to the 45.7% described by park et al. [19] while holding a smartphone during the cpr maneuvers. apparently, no more than 2/3 of the cpr cycles are performed within the recommended range (i.e., between 5.0 and 6.0 cm) [20]. Notably, studies reporting a high cycle ratio within the target depth range (90.9% with a smartphone [21] and 98.7% with a smartwatch [22]) used the manikin’s built-in sensor as the reference. This may emphasize the importance of using a reliable reference system (e.g., a linear variation digital transducer or video) when comparing differences in outcomes.

the group-mean cpr ‘performance’ score achieved in the present study (98.7%) is higher than that reported by other studies (e.g., 89.4 to 91.8% [23]). The higher group-mean score may be attributed to several factors, including the participants being young and physically active. in addition, the CPR was applied during 2-minute bouts with a 2-minute rest/recovery period between cpr bouts, which would have reduced fatigue effects and increased performance consistency. furthermore, participants had undergone recent cpr training, which may have favored obtaining high scores. Finally, the feedback from the mannequin software may have played a role, as it is considered to improve cpr quality performance [20].

The group-average coefficients of variation (CV) for the various cpr parameters determined via smartwatch analysis indicate that participants were more consistent in controlling the temporal aspects (cycle time and frequency; cv ≤ 5.6%) in comparison to the spatial aspects (compression and decompression; cv ≤ 15.6%) (Table 1). This suggests that consistently achieving the appropriate compression depth within a range of 1 cm (5–6 cm) is more challenging than sustaining pacing of 1.6 to 2 cycles per second. This occurred despite the mannequin’s app/software providing real-time feedback regarding both compression depth and cycle frequency. In real emergencies, consistently achieving the recommended compression depth is likely to be much more challenging because of patient-centered differences. for instance, women’s chests are less stiff than men’s, while older adults tend to have stiffer chests than their younger counterparts [17]. Furthermore, it has been demonstrated that applying manual compression for prolonged periods changes the chest’s mechanical properties, requiring compression adjustments over time.

The consequences of performing cpr maneuvers with a deeper compression than the recommended 6 cm are controversial. There are claims that there is insufficient evidence to indicate that excessive compression depth causes damage to the chest [24]. In contrast, others have suggested that excessive compression may produce sternum and rib fractures and associated adverse outcomes [1]. These arguments reinforce the contention that real-time feedback may help ensure cpr effectiveness, especially feedback about appropriate depth compression. In addition, real-time feedback about chest decompression could also improve cpr effectiveness, as allowing complete decompression of the chest after each compression results in better venous return, which is a crucial requirement to facilitate achieving appropriate diastolic pressure [1].

Wearable devices such as smartwatches are attractive, as they have the ability to be linked to a smartphone app to provide detailed and straightforward monitoring of a rescuer’s CPR performance on a cycle-by-cycle (i.e., real-time) basis during training or real-life conditions. They could also be programmed to provide real-time audio feedback (e.g., via a variable ‘beep’ or similar, from the watch itself) regarding CPR performance without the need to be linked to a smartphone. With such app/software and/or hardware developments incorporated in the smartwatch, their use becomes very attractive as they can be worn, which means first aiders/rescuers are not required to hold a recording device (e.g., a smartphone) while applying CPR. as a general rule, compression scores alone provide limited information regarding overall CPR performance [25]. Providing real-time feedback on compression depth, rate, and decompression could improve cpr performance [25, 26]. Thus, developing the smartwatch app/software and/or hardware to provide real-time feedback would allow rescuers or responders to self-regulate their performances and adjust the compression parameters to ensure adherence to cpr guidelines. such should optimize resuscitation efforts and could improve patient survival outcomes [26]. In addition, the app/software could be programmed to indicate the need for a rescuer to ‘swap out’ to allow another rescuer to continue CPR if and when compression parameters are not being maintained due to rescuer fatigue.

The present study showed that smartwatch-derived measures provide fair accuracy while estimating the cpr compression and decompression depth, with a slight overestimation that may be related to the position in which the reference points were defined (e.g., the wrist [smartwatch analysis] vs. the dorsum of the hand [video analysis]). Although measures were taken to reduce possible movement artifact, the flexibility of the carpal and wrist joints may have allowed minor extra displacements at the end of the compression excursion. Some unintended movements of the hand in the frontal plane (e.g., minor hand slippage) may have also influenced the consistency in compression and decompression measurements.

The slight overestimation is comparable to the discrepancies indicated by Song et al. (2016) [14], who compared the measurements obtained from a linear vertical displacement transducer attached to a mannequin’s chest and a smartphone and reported a comparable mean difference (between the two measurement approaches) of 0.23 to 0.53 cm. Although the mean error (i.e., bias) between the smartwatch and the video was around 6%, the limits of agreement were relatively large, resulting in errors up to 23% of the mean target depth (i.e., 5.5 cm). therefore, the use of the present algorithm is relatively limited when estimating compression depth and decompression. future work using different approaches (e.g., artificial intelligence) may improve the algorithm’s accuracy while estimating the displacement-related parameters. unfortunately, most previous studies have only reported the mean differences between measurements regarding compression parameters but omitted the limits of agreement information [9, 14, 15] (i.e., the errors’ spread), and other articles did not perform the analysis and reported on decompression displacement, cycle duration, and cycle time [9–16]. conversely, there was good agreement in the temporal parameters of the CPR when smartwatch and video measures were compared, in which the mean error was negligible (nearly zero), and the limits of agreement (0.05s) revealed that 95% of the data did not exceed 9% of the mean cycle duration (0.53s). therefore, our data indicate that providing feedback on pace is plausible when using a smartwatch.

## Limitations and recommendations

Calculating linear (e.g., vertical) displacement by integrating accelerometer signals faces significant challenges due to noise, bias, and drift, which amplify the errors through multiple integration procedures [27, 28]. Even small constant offsets (bias) in accelerometer readings will cause cumulative drift in velocity and displacement estimates. in the present study, the algorithm used to undertake the double-integration of the acceleration signal to estimate compression depth takes into consideration a number of factors [29]. the first was the adjustments made to the accelerometers’ sampling frequency, which was observed to be inconsistent. Without correcting these time inconsistencies, inaccuracies during the double-integration would grow as the recording time increased. A cycle-by-cycle approach used in the present study may have reduced cumulative errors that usually make long-term displacement estimation unreliable. The error identified when the smartwatch was compared to the video reference system suggests that the present approach is promising while monitoring the rate at which cpr is applied. Despite the evolution of the hardware and processing methods, complete error elimination remains challenging.

The presented study has a number of limitations. The first concerns the position assumed by the participants during the cpr procedures. chest compression experiments were performed on a mannequin placed on hard, flat ground, with the participant ‘first aider’ in a kneeling position. the accuracy of determining CPR compressions might change if the procedures are performed on a mannequin (or patient) placed on a soft bed or reclined chair that would absorb some of the compression force, which could result in a larger compression depth (hand movement) measurement than that being imparted to the chest. Artifact movements may have occurred as the accelerometer cannot account for small flexion/extension that may have occurred in the wrist joint. the compression depth measurements may also be susceptible to changes in the position/posture of the ‘first aider’, e.g., a standing position could change the recorded acceleration signal, which could affect compression depth measurements. the second concern is that the mannequin model did not have the ability to provide cycle-by-cycle data, even though the accuracy of the mannequin’s onboard sensors is not always available. Future work should use a mannequin model that has the ability to monitor and store compression depth and frequency on a cycle-by-cycle basis so that the compression depth and frequency measures determined from analysis of the smartwatch’s acceleration signal could be directly compared. further research is also needed to determine whether the algorithm used to analyze the smartwatch accelerometer signal is robust enough to capture cpr parameters in other conditions (e.g., untrained first-aiders, different rescuer postures, compliant floor conditions, and so on). Other studies are needed to determine if the mean measurement differences between systems (i.e., the measurement bias between smartwatches vs. video or between smartwatches vs. linear variable differential transducer) are constant. once confirmed, this off-set could easily be corrected using a calibration factor in future applications. finally, additional analysis is required to identify if other approaches (i.e., artificial intelligence-based algorithms) may reduce the limits of agreement of the compression depth and decompression.

## Conclusion

Results indicate that analyzing the accelerometer signal from a smartwatch worn by a group of trained ‘first aiders’ enabled assessment of the cpr compression depth and frequency they administered to a mannequin. The limits of agreement between smartwatch-derived cpr parameters and those derived from video analysis indicate that depth and decompression errors are relatively large given the CPR’s target range (5–6 cm). On the other hand, the temporal analysis revealed that the CPR cadence can be accurately measured using the proposed algorithm to process the smartwatch’s acceleration signals.

Further work is needed to develop a smartwatch app that provides real-time feedback to improve cpr performance during training and real-world resuscitation scenarios, especially involving pacing control. such a system may help emergency care.

## Supplementary Information

Below is the link to the electronic supplementary material.

Supplementary Material 1 (XLSX 267 KB)

## Data Availability

The data supporting this study’s findings are available from the corresponding author, JB, upon reasonable request.
